# Small-scale genetic structure of populations of the bulb mite *Rhizoglyphus robini*

**DOI:** 10.1007/s10493-023-00807-1

**Published:** 2023-07-27

**Authors:** Karolina Przesmycka, Jacek Radwan

**Affiliations:** Evolutionary Biology Group, Faculty of Biology, Adam Mickiewicz University, Poznan, Poland

**Keywords:** Acarid mites, Soil, Onions, Pests, Migration, Colonisation, Genetic diversity, Microsatellite loci, Acaridae, Astigmata

## Abstract

Bulb mites are an economically significant pest of subterranean parts of plants and a versatile laboratory animal. However, the genetic structure of their populations remains unknown. To fill this gap in our knowledge of their biology, we set up a field experiment in which we allowed mites to colonize onion bulbs, and then determined the genetic structure of colonisers based on a panel of microsatellite loci. We found moderate but significant population structure among sites separated by ca. 20 m (F_ST_ range 0.03–0.21), with 7% of genetic variance distributed among sites. Allelic richness within some bulbs was nearly as high as that in the total population, suggesting that colonisation of bulbs was not associated with strong population bottlenecks. The significant genetic structure we observed over small spatial scales seems to reflect limited dispersal of mites in soil.

**Supplementary Information:**

The online version contains supplementary material available at 10.1007/s10493-023-00807-1.

## Introduction

Genetic structuring of populations affects key aspects of a species’ biology such as the rate of evolution, local adaptation, population persistence or evolution of social behaviours (Mopper 1996; Gaggiotti 2003; Frean et al. 2013; Débarre et al. 2014; Weiss-Lehman and Shaw 2019). Acarid mites, and particularly *Rhizoglyphus robini*, have been a subject of numerous eco-evolutionary studies (e.g., Lesna and Sabelis 1999; Smallegange and Coulson 2011; Plesnar-Bielak et al. 2012; Skrzynecka and Radwan 2016; Lukasiewicz et al. 2017), yet the genetic structuring of their populations remains unknown. *Rhizoglyphus* is considered a pest of onions and tubers, and thus a subject of considerable applied research (reviewed in Gerson et al. 1991, Diaz et al. 2000), but also due to easiness of rearing in the laboratory and distinct sexually selected male dimorphism it has become a model species in ecological and evolutionary research, with a particular focus on sexual selection. Males of *R. robini* occur in two heritable forms: aggressive heteromorphs (fighters), with a thickened and sharply terminated third pair of legs used for fights, and non-aggressive homeomorphs (scramblers), with unmodified legs (Radwan 1995; Radwan et al. 2000).

The system has been used to demonstrate that, consistent with ‘good genes’ models of sexual selection (Zahavi 1975; Andersson 1986; Rowe and Houle 1996), expression of sexually selected weapons is associated with a lower load of deleterious recessives (Lukasiewicz et al. 2020), and thus their presence helps to purge populations of deleterious mutations and reduce inbreeding depression (Parrett et al. 2022). In poor environments, surviving scramblers were found to be more heterozygous compared to females and fighters at a set of microsatellite loci (Stewart et al. 2019), possibly because under harsh conditions deleterious effects of mutations exposed in homozygous scramblers resulted in early mortality. However, the genes associated with expression of the weapon appear to have negative pleiotropic effect on females fitness (Plesnar-Bielak et al. 2014), illustrating ontogenetic (or intra-locus) sexual conflict (Rice and Chippindale 2001; Bonduriansky and Chenoweth 2009). Another conflict has been detected between male fitness, proportional to the number of mating partners, and the cost of multiple copulations to females (Tilszer et al. 2006). This cost, however, can evolve under kin selection, with male harmfulness to females decreasing when mites evolve in groups of related individuals which then compete globally (Lukasiewicz et al. 2017).

The above results were obtained in experimental evolution studies and laboratory experiments, and their interpolation to natural conditions has been hampered by the lack of knowledge of genetic structure of *R. robini* populations. For example, to appreciate whether sexual conflict in natural populations can be shaped by kin selection, it is necessary to know the extent of genetic structuring of populations (Pizzari et al. 2015). Furthermore, evolution of the occurrence of fights between males can be affected by kin structure of populations. Assuming that individuals can discriminate kin from non-kin, theory predicts that intermale aggression should be non-linearly associated with relatedness, peaking at intermediate values (Reinhold 2003; Gardner and West 2004). This prediction was supported by empirical work on the social spider mite *Stigmaeopsis miscanthi* (Sato et al. 2013), although not in the entomopathogenic nematode *Steinernema longicaudum*, in which there was a monotonic negative relationship between within-group relatedness and aggression (Kapranas et al. 2016). In *R. robini*, relatives tended to be killed in fights less often, although the trend was not significant, and they are less often cannibalised than unrelated individuals (Van den Beuken et al. 2019). *Rhizoglyphus robini* is typically found on bulbs of such plants as onions, garlic, tulips, etc. (Diaz et al. 2000), and it has been speculated that colonisation of bulbs by single gravid females or few individuals can result in most genetic variation being distributed mostly among bulbs, affecting social evolution (Lukasiewicz et al. 2017). Here, by using a panel of microsatellite loci, we investigated this scenario by assessing the fixation coefficient F_ST_, i.e., the measure of inbreeding resulting from genetic similarity of individuals within a deme, compared to that in a total population (Wright 1921; Verity and Nichols 2014).

## Materials and methods

**Fig. 1:**
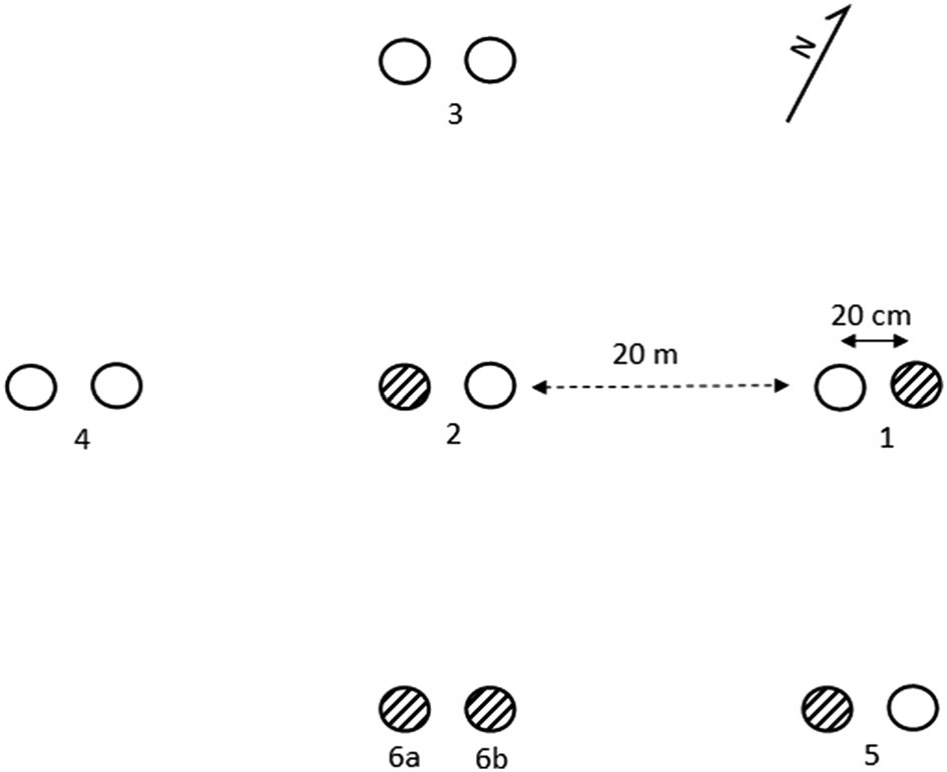
Schematic representation of the placement of onions (circles) in the experimental plot. Each site (numbered 1–6) contained two onions separated by ca. 20 cm. Onions on which mites were found 4 months after they were planted are striped

Onion bulbs obtained from a supermarket were sterilized at 70 °C for 2 h to ensure that any mites which could be present on them would be killed (Gerson et al. 1981). The bulbs were then planted on 30 March 2021 in a small garden plot near Mosina (Poland; 52.213411, 16.922129), at sites separated by ca. 20 m, each site containing two onions separated by ca. 20 cm, as shown in Fig. 1. Their locations were marked with flagged sticks. The plot served as a vegetable garden and has not been fertilised for at least the last 5 years. We previously collected bulb mites at this location from hyacinth bulbs. It is bordered by grass lawns and an orchard (W). Further away to the NE, it is bordered by sparsely distributed houses, and then 20 m away a gravel road and the Warta river (ca. 50 m wide). There are arable fields in all other directions within 50–100 m, but separated from the plot by a 100-m stretch of wasteland (NW), tarmac road (W) and farm buildings and a narrow dirt road (SE). On 23 June 2021, the onions were removed from the soil, packed each in a separate container, and brought to the laboratory where they were thoroughly examined for the presence of mites under an Olympus SZ10 stereomicroscope. The collected mites were preserved in 97% analytical ethanol and stored at −20 °C. Prior to DNA extraction, mites were homogenised and incubated in 180 µl ATL buffer (Qiagen) and 20 µl proteinase K in 56 °C for 22 h. DNA was extracted from 21 to 30 adult mites per onion following QIAamp DNA Mini and Blood Mini (Qiagen) protocol.

Twelve microsatellite loci were then amplified in four multiplex PCRs using primers designed by Kolasa (2015) (Stewart et al. 2019). The 10 µl PCR mixture contained: 1 µl of approximately 0.3–3.0 ng/µl of DNA, 5 µl of 2× QIAGEN Multiplex PCR Master Mix, 0.1–0.2 µM of forward primer with specific M13 tail, 0.2–0.4 µM of reverse primer and 0.2–0.4 µM of fluorescently labelled specific M13 primer (Table S1). Thermal conditions for PCRs were: 15 min of initial denaturation at 95 °C; 35 cycles of 30 s at 94 °C, 60 s at 55 °C, and 60 s at 72 °C; and a final extension of 72 °C for 10 min. PCR products were separated and sized on an 3130xl Genetic Analyser (Applied Biosystems) using Liz 600 size standard. Genotyping was performed in GeneMapper v.3.7 (Applied Biosystems).

In all analyses mites found on one onion were treated as a population. All loci in each population were checked for deviations from Hardy–Weinberg expectations and for linkage disequilibrium between them using GENEPOP v.4.7.5 (Rousset 2008). The presence of null alleles was tested using the algorithm of Dempster et al. (1977) implemented in FreeNA (Chapuis and Estoup 2007). Bonferroni-Holm correction (Holm 1979) was applied to correct for multiple testing in both of the above analyses. Allelic richness of each locus and population was calculated using the R (R Core Team 2016) package *popgenreport* (Adamack and Gruber 2014). Pairwise F_ST_ values, their 95% confidence intervals and AMOVA were calculated using the R package *hierfstat* (Goudet 2005).

## Results

Mites colonized five out of 14 onions set up (Fig. 1). We extracted DNA and successfully genotyped 21–30 mites per population. Ten out of 12 alleles were polymorphic (Table S1). The number of microsatellite alleles per locus ranged from 3 to 10 (mean 5.1). After correcting for multiple comparisons, significant deviation from Hardy–Weinberg equilibrium was detected in 6/50 (12%) tests (Table S2). Three of these cases were for Rr40, which was likely due to the presence of null alleles, detected in four populations at this locus (except population 5) at frequencies 0.12–0.28. The other three loci (Rr51, Rr79, Rr91) showed deviation from HW only in population 1, and in all three cases F_IS_ was negative, indicating excess of heterozygotes. Evidence for null alleles occurring at frequency > 0.1 was found at five additional loci, but in addition to Rr40 this pattern was repeatable across populations only at loci Rr47 (3/5 populations, frequencies 0.12–0.15), Rr91 (3/5 populations, frequencies 0.08–0.15) and Rr92 (3/5 populations, frequencies 0.09–0.13). Allelic richness for all loci and populations are shown in Table 1. Populations in the SW part of the plot (2, 6a and 6b) showed higher allelic richness compared to those from the NE part (1, 5).

**Table 1:** Allelic richness for all loci (Rr03–Rr092) across populations (1–6b)

	1	2	5	6a	6b
Rr03	2.917	2.000	2.000	2.865	2.753
Rr31	1.999	2.995	2.000	2.986	2.999
Rr40	2.707	4.815	2.686	7.501	6.992
Rr47	3.624	3.971	2.686	3.978	3.941
Rr51	3.917	3.961	3.000	3.952	2.997
Rr61	3.414	3.991	2.999	4.960	4.929
Rr72	2.000	3.815	1.000	1.983	2.695
Rr79	3.917	3.961	3.000	3.965	2.997
Rr91	3.994	7.644	2.000	7.430	6.995
Rr92	1.000	1.972	1.686	2.978	2.997

Linkage disequilibrium was detected in 20/227 (8%) tests (Table S4). Most of these LDs were repeatable between populations (Rr51–Rr79 in all five populations, Rr47–Rr91 3/5 populations, Rr47–Rr40 3/5 and Rr40–Rr91 4/5), indicating a possibility of two physical linkage groups (Rr51–Rr79 and Rr40–Rr47–Rr91). We therefore retained for further analyses only one locus per linkage group, based on criteria of higher polymorphism, fewer missing genotypes, and lower proportion of null alleles. We thus rejected from further analyses loci Rr40, Rr47 and Rr79. However, results of analyses of such a selected subset of seven loci that we report below were very similar to those based on the full set of 10 loci (not shown).

Pairwise F_ST_ values among populations are given in Table 2. They ranged between 0.03 and 0.2 between population isolated by ca. 20 m (Fig. 1). Confidence intervals of all F_ST_ values except for the lowest of these (between populations 2 and 6a) did not overlap zero. F_ST_ among the neighbouring populations 6a and 6b (isolated by ca. 20 cm) was only 0.014 (95% CI = −0.001–0.035; Table 2). Accordingly, AMOVA showed that a significant portion of variance was distributed among sites (7%, P = 0.03), whereas the differentiation between onions within a site (2%) was not significant (P = 0.18).

**Table 2:** Pairwise F_ST_ values and their 95% confidence intervals

	1	2	5	6a	6b
1	NA	0.09 (0.03–0.15)	0.21 (0.11–0.31)	0.10 (0.06–0.14)	0.12 (0.08–0.17)
2		NA	0.08 (0.04–0.10)	0.03 (0.00–0.06)	0.06 (0.02–0.10)
5			NA	0.07 (0.02–0.14)	0.09 (0.01–0.18)
6a				NA	0.01 (0.00–0.04)
6b					NA

## Discussion

In this study we determined small-scale genetic differentiation between bulb mite populations inhabiting separate onions. We found significant, but moderate, differentiation between onions between populations at sites separated by 20 m. However, no significant differentiation was detected within sites, i.e., among bulbs separated by 20 cm, although the confidence interval for the F_ST_ estimate only marginally overlapped zero. Likewise, AMOVA showed that a significant portion of genetic variation was explained by differentiation between sites, but not between bulbs within the site. To our knowledge, our study is the first that investigated population structure of bulb mites in the wild.

Genetic differentiation at a spatial scale similar to ours was examined in other mite taxa. Navajas et al. (2002) studied genetic structure in the spider mite *Tetranychus urticae* population in a greenhouse, and found no significant genetic differentiation between mites collected from different plants, with F_ST_ estimates ranging between 0.008 and 0.09. In the eriophyid blister mite (*Colomerus vitis*) there was moderate, but significant differentiation among populations inhabiting patches of vines located in the same plot (F = 0.089, P = 0.012) (Carew et al. 2004). However, we know of no study that looked on small-scale population structure of mites inhabiting subterranean parts of plants.

Our study is consistent with the results from experiments using garlic traps in arable fields in Israel, based on which Gerson et al. (1985) concluded that mites persistently inhabit soils and relatively easily colonise bulbs planted in these soils. However, our results indicate that even at small spatial scale of 20 m the colonisation can result in significant genetic structuring of population. The structuring was not extreme, and explained only 7% of the genetic variation, most of which resided within bulbs. This indicates that bulbs were unlikely to be colonised by single gravid females or very few mites. Indeed, in some populations (6a, 6b, and 2), allelic richness within a bulb was nearly as high as that in the total population, suggesting that the bulbs were colonised by at least several mites.

Colonisation by several mites implies that a single onion will contain both relatives (progeny of the same parents) and unrelated individuals. This could select for kin recognition abilities, that can be used to avoid killing and cannibalising relatives (Van den Beuken et al. 2019), or mating with relatives to avoid inbreeding depression (Pike et al. 2021). This latter possibility remains to be investigated in the bulb mite. Moderate, although significant, genetic structuring among bulbs implies that kin selection for costly traits benefiting group members, such as reduced harm to females at the cost to male reproductive success (Wild et al. 2011; Lukasiewicz et al. 2017), may be limited in bulb mite populations. This may potentially explain persistence of sexual conflict in the bulb mite (Kolodziejczyk and Radwan 2003; Tilszer et al. 2006). However, adjusting the degree of male harm in relation to his relatedness to group members (Carazo et al. 2014) remains a possibility worth investigating in this species.

The significant genetic structure we observed seems instead to reflect limited dispersal of mites to some areas of the plot, which is consistent with the fact that not all onions were colonised. Populations from the NE part of the plot (1 and 5) had lower allelic richness, and exhibited the highest F_ST_ values between them. This indicates that they were colonised by fewer individuals, which resulted in their higher genetic differentiation. This may reflect smaller mite densities in the soil where onions 1 and 5 were planted. The reasons for these presumed differences in mite density in our plot are not known but may be due to the history of vegetables planted on the plot, and/or the quality/humidity of soil (Gerson et al. 1985). Some limited inflow of mites from nearby arable fields near the SW part of the plot could not be excluded, particularly from the nearest field at the SE separated only by a narrow dirt road, which contribute to differences in mite genetic variation within the plot studied.

We acknowledge that our results on the genetic structure between onions within sites are at best provisional, as we only recovered mites on one such pair of onions separated by 20 cm. The F_ST_ estimate is the lowest among the ones we obtained and not significant, which could be a result of migration from one bulb to the next, or due to colonisation of both onions from the same source population inhabiting local soil. The former possibility predicts that allelic composition of the recipient onion should be a subset of that present in the source onion, but although we observed some allele sharing between 6a and 6b, private alleles were present on both (e.g., allele 151 at Rr51, allele 150 at Rr31 and allele 167 at Rr71 in 6a, and allele 154 at Rr31 and 140 at Rr61 in 6b). This pattern is more consistent with the latter possibility, i.e., independent colonisation of the neighbouring onions from the same source population residing in local soil. This additionally highlights the role of soil as a reservoir of mites colonising bulbs and tubers (Gerson et al. 1985).

Given the role of soil as reservoir and dispersal medium, genetic structuring of bulb mite populations may depend on soil properties determining mite persistence and migration patterns. In soil rich with nutrients, mites may survive and subsequently colonise more productive habitat patches such as bulbs of garlic or onions (Gerson et al. 1985). However, in more adverse soils (few nutrients, low humidity; see Gerson et al. 1985), persistence may be limited. Therefore, genetic structuring, and thus its role in shaping evolution of social behaviour (West et al. 2002; Débarre et al. 2014) or population persistence (Weiss-Lehman and Shaw 2019) of bulb mites may depend on the quality of soil constituting the medium of mite persistence and migration. More work is therefore needed that would cover a broader range of soils and environments inhabited by bulb mites.

## Supplementary Information

Below is the link to the electronic supplementary material.

Supplementary material 1 (DOCX 50.7 kb)
