# Supplementary material for: Small-scale genetic structure of populations of the bulb mite Rhizoglyphus robini
Source: Exp Appl Acarol. 2023 Jul 27;90(3-4):219–26. doi: 10.1007/s10493-023-00807-1 (PMC10406659; doi:10.1007/s10493-023-00807-1)
Supplement: Supplementary file 1 — Supplementary material 1 (DOCX 50.7 kb) [file 10493_2023_807_MOESM1_ESM.docx]

**Table S1.** Primers used for the microsatellite loci amplification. Specified are primer sequences (F, forward, R, reverse), PCR multiplexes (Mix) in which primers were amplified, size range (Range), number of alleles found (N.als.), primer concentration used in PCR (µM per reaction) and associated fluorescent dye.

| Locus | Primer sequence (5' - 3') | Mix | Range  (bp) | N.als | Primer  conc.  (µM) | Fluoresc. dye |
| --- | --- | --- | --- | --- | --- | --- |
| **Rrms03** | F: AACTTGGTCTAAAGTGAAGCA | 4 | 159-169 | 3 | 0.1 | VIC |
|  | R: TTGAAAAGTCACTAAGCCAAC |  |  |  | 0.2 |  |
| **Rrms18** | F: GCTTTCATTGTTGTACACCTC | 1 | NA | NA | 0.1 | 6FAM |
|  | R:ACAAACAGCAATGAGGTACAG |  |  |  | 0.2 |  |
| **Rrms23** | F: CCGTAATGTACGACAAAGTGT | 1 | NA | NA | 0.1 | PET |
|  | R: AAGGTAATCTATCCCCCACT |  |  |  | 0.2 |  |
| **Rrms31** | F: GATATTGGCGAATAGCTCAC | 2 | 148-154 | 3 | 0.1 | 6FAM |
|  | R: TACTCGGTCAAGTCAATTCTC |  |  |  | 0.2 |  |
| **Rrms40** | F: GTAATGGCCATGTCACTAGC | 4 | 132-164 | 8 | 0.1 | 6FAM |
|  | R: TTTGAGACTCGAAAGAAACAG |  |  |  | 0.2 |  |
| **Rrms47** | F: TCTTTCCTTCAGTACTCGTCA | 2 | 131-149 | 5 | 0.1 | PET |
|  | R: GCATAATTGTGCATGTGTGT |  |  |  | 0.2 |  |
| **Rrms51** | F: TCTTACTCACATTGGTTGCTT | 1 | 139-159 | 5 | 0.1 | VIC |
|  | R: CTGTGCATGTTGGCTAATAAT |  |  |  | 0.2 |  |
| **Rrms61** | F: TAAATAGATCGAGACGACCAA | 3 | 112-140 | 5 | 0.1 | PET |
|  | R: TCTCTGTGTGAACGATCTGTA |  |  |  | 0.2 |  |
| **Rrms72** | F: GAAATGTCAAAGACGAAAGTG | 4 | 155-167 | 4 | 0.1 | PET |
|  | R: TTGAAGTGCGAAATTAGTCAT |  |  |  | 0.2 |  |
| **Rrms79** | F: TCTTACTCACATTGGTTGCTT | 2 | 139-159 | 5 | 0.1 | VIC |
|  | R: CTGTGCATGTTGGCTAATAAT |  |  |  | 0.2 |  |
| **Rrms91** | CTATGTTGAAAAGGCATCAAT | 3 | 105-151 | 10 | 0.1 | VIC |
|  | GCAAAGTGTTGTTCACTCAAT |  |  |  | 0.2 |  |
| **Rrms92** | TTACCGATTAGTTACGTTTGC | 3 | 130-152 | 3 | 0.1 | 6FAM |
|  | ATTCACATTATCCGCATCTAA |  |  |  | 0.2 |  |

Table S2. Weir & Cockerham's F_IS_ estimates for the five populations across microsatellite loc. Deviations from Hardy-Weinberg equilibrium with p-values significant after Bonferroni-Holm correction (see methods) are indicated in bold.

| Locus | Population | | | | |
| --- | --- | --- | --- | --- | --- |
|  | 1 | 2 | 5 | 6a | 6b |
| Rr03 | -0.386 | -0.276 | -0.393 | -0.270 | -0.0784 |
| Rr31 | -0.189 | -0.197 | 0.071 | 0.110 | -0.0329 |
| Rr40 | **0.836** | 0.274 | **-0.760** | 0.307 | **0.3931** |
| Rr47 | -0.147 | 0.363 | -0.039 | 0.310 | 0.4234 |
| Rr51 | **-0.501** | -0.155 | -0.236 | -0.269 | 0.0400 |
| Rr61 | -0.243 | 0.051 | -0.248 | -0.130 | 0.0988 |
| Rr72 | **-0.466** | -0.122 | NA | -0.061 | -0.0345 |
| Rr79 | -0.501 | -0.155 | -0.236 | -0.163 | 0.0400 |
| Rr91 | **-0.257** | 0.198 | 0.019 | 0.206 | 0.3443 |
| Rr92 | NA | 0.477 | NA | 0.381 | 0.387 |

Table S3. Null allele frequencies for all loci (Rr03-Rr092) across populations (1-6b) estimated using Dempster algorithm implemented in FreeNA.

|  | **Rr03** | **Rr31** | **Rr40** | **Rr47** | **Rr51** | **Rr61** | **Rr72** | **Rr79** | **Rr91** | **Rr92** |
| --- | --- | --- | --- | --- | --- | --- | --- | --- | --- | --- |
| **1** | 0.0000 | 0.0000 | 0.2794 | 0.0000 | 0.0000 | 0.0000 | 0.0000 | 0.0000 | 0.0000 | 0.0010 |
| **2** | 0.0000 | 0.0000 | 0.1226 | 0.1376 | 0.0000 | 0.0000 | 0.0000 | 0.0000 | 0.1002 | 0.0955 |
| **5** | 0.0000 | 0.0166 | 0.0000 | 0.0000 | 0.0000 | 0.0000 | 0.0010 | 0.0000 | 0.0003 | 0.0000 |
| **6a** | 0.0000 | 0.0597 | 0.1219 | 0.1244 | 0.0000 | 0.0000 | 0.0000 | 0.0000 | 0.0844 | 0.1175 |
| **6b** | 0.0000 | 0.0000 | 0.1742 | 0.1531 | 0.0000 | 0.0324 | 0.0000 | 0.0000 | 0.1501 | 0.1353 |

Table S4. Χ^2^ test for lineage disequilibria between pairs of loci

| Pair | Χ^2^ | df | P |
| --- | --- | --- | --- |
| Rr51 & Rr31 | 10.103 | 10 | 0.431 |
| Rr51 & Rr47 | 23.316 | 10 | 0.009 |
| Rr31 & Rr47 | 23.501 | 10 | 0.009 |
| Rr51 & Rr79 | >159.42 | 10 | <0.001 |
| Rr31 & Rr79 | 9.855 | 10 | 0.453 |
| Rr47 & Rr79 | 19.723 | 10 | 0.031 |
| Rr51 & Rr61 | 15.612 | 10 | 0.111 |
| Rr31 & Rr61 | 5.186 | 10 | 0.878 |
| Rr47 & Rr61 | 11.480 | 10 | 0.321 |
| Rr79 & Rr61 | 15.076 | 10 | 0.129 |
| Rr51 & Rr91 | 15.621 | 10 | 0.110 |
| Rr31 & Rr91 | 16.654 | 10 | 0.082 |
| Rr47 & Rr91 | >116.93 | 10 | <0.001 |
| Rr79 & Rr91 | 15.687 | 10 | 0.108 |
| Rr61 & Rr91 | 18.729 | 10 | 0.043 |
| Rr51 & Rr92 | 7.285 | 8 | 0.506 |
| Rr31 & Rr92 | 11.156 | 8 | 0.192 |
| Rr47 & Rr92 | >59.257 | 8 | <0.001 |
| Rr79 & Rr92 | 7.198 | 8 | 0.515 |
| Rr61 & Rr92 | 10.770 | 8 | 0.215 |
| Rr91 & Rr92 | 23.290 | 8 | 0.003 |
| Rr51 & Rr03 | 11.850 | 10 | 0.295 |
| Rr31 & Rr03 | 5.281 | 10 | 0.871 |
| Rr47 & Rr03 | 12.486 | 10 | 0.253 |
| Rr79 & Rr03 | 12.245 | 10 | 0.268 |
| Rr61 & Rr03 | 11.175 | 10 | 0.344 |
| Rr91 & Rr03 | 21.604 | 10 | 0.017 |
| Rr92 & Rr03 | 7.824 | 8 | 0.450 |
| Rr51 & Rr40 | 17.152 | 10 | 0.071 |
| Rr31 & Rr40 | 17.625 | 10 | 0.061 |
| Rr47 & Rr40 | >88.958 | 10 | <0.001 |
| Rr79 & Rr40 | 18.207 | 10 | 0.051 |
| Rr61 & Rr40 | 8.074 | 10 | 0.621 |
| Rr91 & Rr40 | >110.76 | 10 | <0.001 |
| Rr92 & Rr40 | 32.143 | 8 | <0.001 |
| Rr03 & Rr40 | 13.424 | 10 | 0.200 |
| Rr51 & Rr72 | 7.246 | 8 | 0.510 |
| Rr31 & Rr72 | 5.102 | 8 | 0.746 |
| Rr47 & Rr72 | 6.844 | 8 | 0.553 |
| Rr79 & Rr72 | 5.472 | 8 | 0.706 |
| Rr61 & Rr72 | 3.808 | 8 | 0.873 |
| Rr91 & Rr72 | 9.135 | 8 | 0.331 |
| Rr92 & Rr72 | 7.335 | 6 | 0.290 |
| Rr03 & Rr72 | 11.062 | 8 | 0.198 |
| Rr40 & Rr72 | 8.182 | 8 | 0.415 |
